# Neuroendocrine cells signal for repair: hedgehog leading the way

**DOI:** 10.1038/s41392-025-02386-6

**Published:** 2025-09-11

**Authors:** Mengjie Li, Mengmeng Zhou, Jin Jiang

**Affiliations:** 1 Department of Molecular Biology, University of Texas Southwestern Medical Center, Dallas, TX 75390 USA; 2 Department of Pharmacology, University of Texas Southwestern Medical Center, Dallas, TX 75390 USA

**Keywords:** Regeneration, Disease model

A recent study published in *Cell*^1^ revealed that Desert hedgehog (Dhh) produced by neuroendocrine cells initiates a feedback response from underlying stromal cells to orchestrate a widespread repair of injured airway epithelia or pancreatic islets, revealing a common role of epithelial-stromal feedback signaling in promoting tissue repair.

The mammalian respiratory system is responsible for gas exchange, making it vulnerable to injury from inhaled pollutants or respiratory viruses; yet the mechanisms that promote tissue repair to preserve adult airway integrity have remained poorly understood. The Hedgehog (Hh) signaling pathway governs embryonic development and adult tissue homeostasis.^2^ In organs like the mouse urinary bladder, epithelium-derived Hh signals trigger a feedback response from stromal cells to regulate epithelial homeostasis and repair.^3^ To investigate the involvement of Hh signaling in adult tracheal airway repair, the authors examined the steady-state expression of Hh ligands. Surprisingly, among the three Hh family members, only Dhh exhibited detectable expression by RNA-seq, whereas Sonic hedgehog (Shh) and Indian hedgehog (Ihh) were undetectable. Importantly, the expression of a pathway reporter *Gli1*—encoding a member of the Gli-family of Hh pathway transcription factors—in stromal cells was diminished in *Dhh*^*−/−*^ mice, suggesting that Dhh is the major Hh ligand responsible for activating *Gli1* in adult tracheal airway. To determine whether Dhh is essential for airway regeneration after injury, the authors examined mice subjected to inhalation of SO_2_, which induces damage of proximal airway epithelial cells. They found that 1) SO_2_ caused more dramatic epithelial damage in *Dhh*^*−/−*^ mice than in control mice, and 2) *Gli1*^*−/−*^ mice suffered severe epithelial damage like *Dhh*^*−/−*^ mice, suggesting that Dhh promotes airway epithelial repair through Gli1.

Next, the authors introduced a tamoxifen-inducible Cre recombinase (*CreERT2*) into the *Dhh* locus (*Dhh*^*CreERT2*^) to mark *Dhh*-expressing cells. They found that Dhh is only expressed in sparsely spaced pulmonary neuroendocrine cells along the airway epithelium during homeostasis. Importantly, ablating *Dhh*-expressing neuroendocrine cells by diphtheria toxin in *Dhh*^*CreERT2*/+^; *Rosa26*^*DTR/DTR*^ mice phenocopied *Dhh*^−/−^ mice with respect to the defect in injury response. Inactivation of the Hh signal transducer Smoothened (Smo) from *Gli1*-expressing stromal cells generated nearly identical phenotypes, suggesting that neuroendocrine cell-derived Dhh acts through Smo in Gli1^+^ stromal cells to promote tissue repair.

**Fig. 1:**
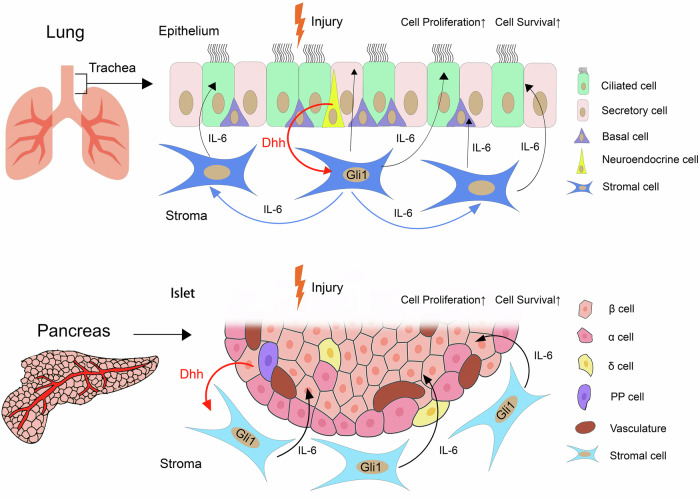
Dhh-initiated epithelial-mesenchymal feedback (EMF) signaling regulates tissue repair in lung and pancreas. In response to injury, neuroendocrine cells in the tracheal epithelium of the lung produce Dhh (red arrow) that stimulates the expression of IL-6 in neighboring Gli1^+^ stromal cells. IL-6 then signals back to promote a widespread repair of the airway epithelium (black arrows). The expression of *IL-6* initiates in Gli1^+^ mesenchymal cells and then propagates to non-Gli1-expressing cells (blue arrows), leading to EMF signal amplification. In the pancreas, Dhh derived from islet β cells induces the production of IL-6 in Gli1^+^ mesenchymal cells, which protects β cells from injury. The illustrations were generated using Procreate and Adobe Photoshop

To identify the downstream mediators, the authors carried out RNAseq analysis of the tracheal airway in mice exposed to SO_2_ and found that, among all the cytokines/growth factors, Interleukin-6 (IL-6) is the most upregulated one dependent on Dhh and Gli1. Importantly, *IL-6*^−/−^ mice suffered epithelial damage as severe as *Dhh*^−/−^ or *Gli1*^−/−^ mice, and topical administration of an IL-6 receptor blocking antibody caused similar epithelial damage. Further analysis revealed that the expression of *IL-6* was initially restricted in Gli1^+^ stromal cells after injury and then propagated into non-Gli1-expressing cells. Hence, upon injury, neuroendocrine cell-derived Dhh induces *Gli1*-expressing stromal cells to produce IL-6 that propagates to the entire tracheal epithelium to ameliorate tissue damage. This epithelial-mesenchymal feedback (EMF) loop amplifies the initially weak signals originating from the rare neuroendocrine cells, resulting in a widespread regenerative response that promotes cell proliferation and survival (Fig. 1).

To extend their findings beyond chemical injury, the authors investigated the role of EMF signaling in injury caused by infection with respiratory viruses such as the H1N1 strain of influenza A and the WA-1 strain of SARS-CoV-2. They found that H1N1 severely injured the airway epithelia in *Gli1*^*−/−*^ mice, whereas a Smo agonist SAG21k protected against airway injury from H1N1 infection. On the other hand, blockage of Hh signaling by a Smo inhibitor XL-139 exacerbated the airway damage caused by WA-1 infection, suggesting that EMF exerts a protective function against infection-induced epithelial damage.

To determine whether EMF signaling initiated from neuroendocrine cells plays a more general role in damage response, the authors turned to the pancreas. They found that >95% of Dhh^+^ cells are β endocrine cells, whereas *Gli1*-expressing cells appear to be islet mesenchymal support cells. Streptozotocin (STZ), a naturally occurring compound particularly toxic to β cells, induced *IL-6* expression in Gli1^+^ cells dependent on Dhh and Gli1. STZ treatment resulted in more dramatic loss of β cells and consequently higher blood glucose levels in *Dhh*^*−/−*^ mice than in control mice, and these defects could be ameliorated by SAG21k treatment, suggesting that augmentation of EMF signaling activity might benefit diabetic patients caused by β cell loss.

Finally, to corroborate the physiological relevance of EMF in humans, the authors analyzed two independent cohorts of 90 and 251 patients treated with FDA-approved SMO antagonists (vismodegib, sonidegib, or glasdegib) for basal cell carcinoma (BCC). BCC patients treated with Hh-pathway inhibitors exhibited a 1.89-fold increased incidence of diabetes compared to the untreated cohorts, suggesting that inhibition of Hh signaling may compromise β-cell integrity and function in BCC patients.

Taken together, this elegant and comprehensive study uncovered a novel function of neuroendocrine cells and Dhh-initiated EMF signaling in acute injury response in adult mouse tracheal epithelium and pancreatic islets (Fig. 1). Coupled with previous findings that EMF also regulates the homeostasis and regeneration of other organs in mice,^4^ these results suggest that EMF signaling may play a more general role in organ regeneration.

Like many discoveries, this study also raises many important questions. e.g., why is Dhh only expressed in neuroendocrine cells? Does this relate to the normal functions of neuroendocrine cells, such as injury sensing? How does injury intersect with the EMF pathway? The author noticed that injury did not affect the expression of *Dhh* and *Gli1* but rather increases the binding of Gli1 on the promoter/enhancer region of *IL-6*, suggesting that injury may act through an unknown epigenetic factor(s) or coactivator(s) to modulate Gli1 activity. It remains to be determined whether these new findings are applicable to other neuroendocrine cell-bearing organs in mice and whether they can be extended to human organs. Finally, although the study suggests that modulating EMF by Hh pathway agonists may have beneficial effects in ameliorating airway or islet injury, care must be taken because excessive Hh signaling might lead to bronchopulmonary dysplasia and chronic obstructive pulmonary disease^5^ as well as cancer.
